# Context‐Dependent Roles of ANGPTL2‐Mediated Inflammaging in Tissue Homeostasis, Pathological Tissue Remodeling, and Longevity

**DOI:** 10.1111/acel.70370

**Published:** 2026-01-08

**Authors:** Shinsei Yumoto, Haruki Horiguchi, Keishi Miyata, Tsuyoshi Kadomatsu, Zhe Tian, Michio Sato, Kimi Araki, Masaaki Iwatsuki, Yuichi Oike

**Affiliations:** ^1^ Department of Molecular Genetics, Graduate School of Medical Sciences Kumamoto University Kumamoto Japan; ^2^ Department of Gastroenterological Surgery, Graduate School of Medical Sciences Kumamoto University Kumamoto Japan; ^3^ Department of Aging and Geriatric Medicine, Graduate School of Medical Sciences Kumamoto University Kumamoto Japan; ^4^ Center for Metabolic Regulation of Healthy Aging (CMHA), Graduate School of Medical Sciences Kumamoto University Kumamoto Japan; ^5^ Division of Developmental Genetics Institute of Resource Development and Analysis, Kumamoto University Kumamoto Japan

**Keywords:** ANGPTL2, inflammation, longevity, SASP

## Abstract

Chronic inflammation is a key driver of aging‐related diseases, obesity‐associated metabolic disorders, and tumor progression. Aging and obesity contribute to the accumulation of senescent cells, which secrete senescence‐associated secretory phenotype (SASP) factors that promote tissue remodeling and chronic inflammation. Here, we investigated the pathological roles of angiopoietin‐like protein 2 (ANGPTL2), a potential SASP factor, in a mouse model of high‐fat diet‐induced premature aging. We found that ANGPTL2 deficiency shortened lifespan but attenuated systemic inflammation, indicating a complex role for ANGPTL2 in aging‐related processes. ANGPTL2 was required for maintaining intestinal homeostasis under metabolic stress; however, ANGPTL2 also exacerbated adipocyte hypertrophy and cardiac dysfunction. Furthermore, ANGPTL2‐mediated inflammation promoted kidney fibrosis but paradoxically protected against perivascular fibrosis in the liver, indicating its organ‐specific effects on fibrotic remodeling. In addition, ANGPTL2 influenced immune responses by driving bronchus‐associated lymphoid tissue formation. These findings suggest that ANGPTL2 has context‐dependent effects, balancing tissue homeostasis and inflammation‐driven pathologies. Our study provides novel insights into the dual roles of ANGPTL2 as a SASP factor in regulating inflammation, fibrosis, and tissue remodeling across different organ systems.

## Graphical abstract

Yumoto et al. show that ANGPTL2 regulates inflammation, fibrosis, and tissue remodeling in high‐fat diet‐induced premature aging. ANGPTL2 deficiency shortens lifespan but reduces inflammation, revealing its context‐dependent and organ‐specific roles in maintaining homeostasis.

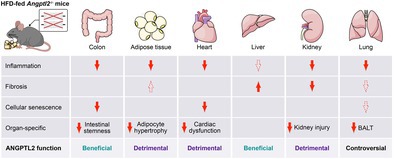

## 1 Introduction

Inflammation is a fundamental biological response that serves as a crucial defense mechanism, maintaining homeostasis in response to injury or infection. While acute inflammation is essential for host defense and tissue repair, chronic inflammation contributes to the development and progression of metabolic disorders, cardiovascular diseases, and even some cancers (Furman et al. 2019). Notably, aging‐related systemic chronic inflammation, termed “inflammaging,” is a key driver of age‐related diseases (López‐Otín et al. 2023; Franceschi et al. 2018). One proposed mechanism underlying inflammaging involves the accumulation of senescent cells, which secrete inflammatory cytokines, chemokines, and growth factors, an activity described as the senescence‐associated secretory phenotype (SASP) (Di Micco et al. 2021). Because SASP factors can promote chronic inflammation, the selective elimination of senescent cells, known as senolysis, has been proposed to antagonize age‐related diseases (Chaib et al. 2022). However, SASP factors also play critical physiological roles, including immune cell recruitment, tissue regeneration, and even embryonic development (de Magalhães 2024). Therefore, a deeper understanding of the dual roles of SASP factors, both beneficial and detrimental, is essential for optimizing targeted senolytic therapies.

Previously, we and others identified a family of secretory proteins structurally similar to angiopoietins, termed angiopoietin‐like proteins (ANGPTLs) (Oike et al. 2003; Hato et al. 2008). Among them, ANGPTL2 has been identified as a potential SASP factor (Thorin‐Trescases et al. 2021), given that its systemic levels reportedly increase with age (Horio et al. 2014). ANGPTL2 promotes adaptive inflammation and facilitates tissue regeneration under physiological conditions, whereas excessive ANGPTL2 activation in response to prolonged stress leads to chronic inflammation and irreversible tissue remodeling, disrupting tissue homeostasis (Kadomatsu et al. 2014). These maladaptive effects of ANGPTL2 promote the pathogenesis of various age‐related diseases, including metabolic disorders, cardiovascular diseases, and possibly some cancers (Kadomatsu et al. 2014). Given these findings, inhibiting ANGPTL2 activity has been proposed as a potential anti‐aging intervention.

Here, we investigate the role of ANGPTL2 in inflammation, tissue remodeling, and lifespan regulation. Our findings demonstrate that ANGPTL2 is a critical factor for longevity in a mouse model of premature aging and functions as a multifaceted regulator of inflammatory responses, tissue remodeling, and fibrosis. Importantly, we reveal the complex nature of ANGPTL2‐mediated inflammation, particularly in the context of intercellular crosstalk across multiple tissues. Our findings provide novel insights into the intricate balance between beneficial and detrimental inflammation mediated by ANGPTL2.

## 2 Results

### 2.1 ANGPTL2 Deficiency Accelerates Premature Aging Induced by a High‐Fat Diet

**FIGURE 1:**
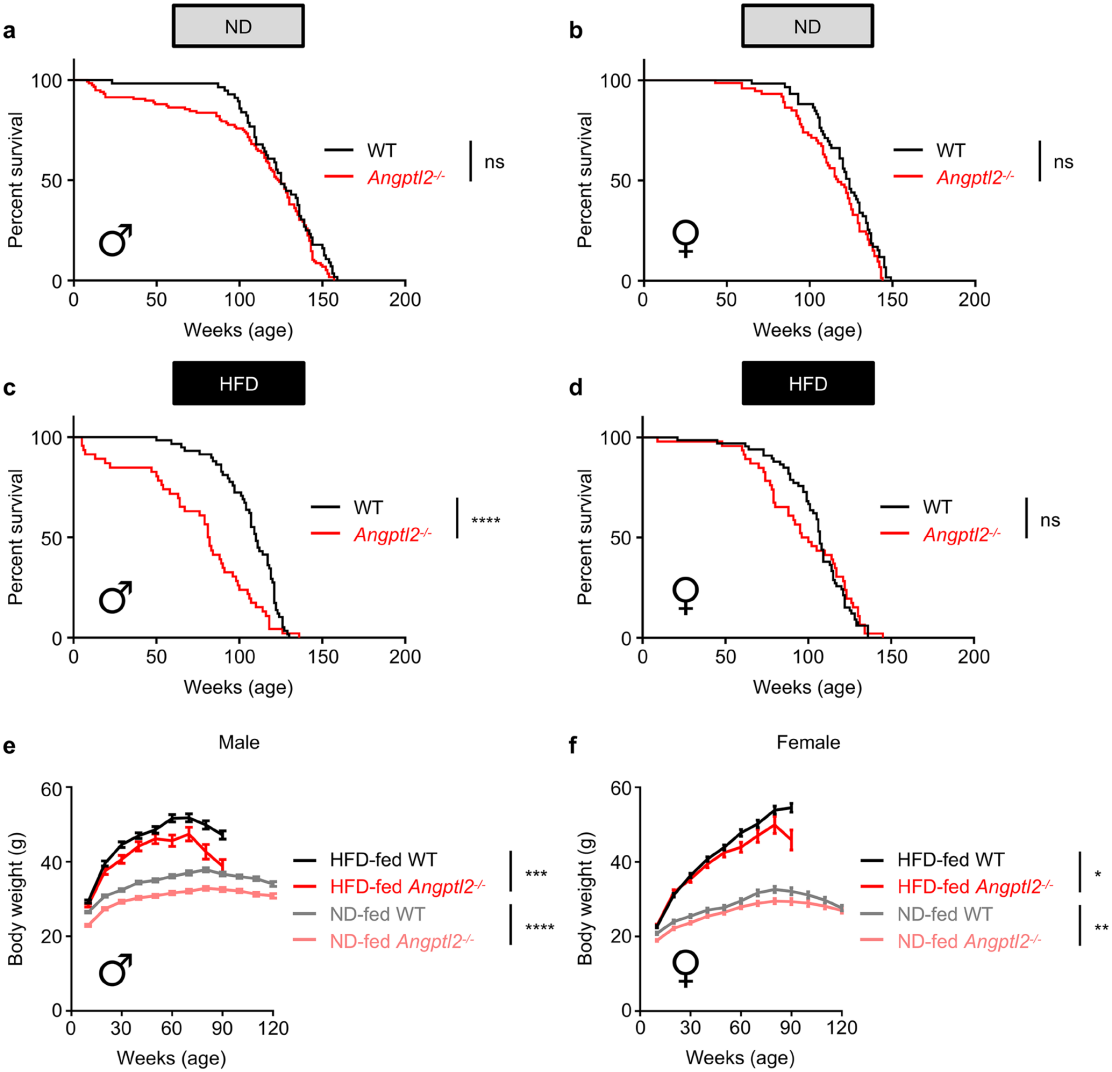
ANGPTL2 deficiency shortens lifespan in a mouse model of high‐fat diet‐induced premature aging. WT and *Angptl2* ^−/−^ mice were fed an HFD, starting at 6 weeks of age. (a) Kaplan–Meier survival curves of ND‐fed male WT (*n* = 56) and ND‐fed male *Angptl2* ^−/−^ (*n* = 116) mice. ns, not significant (*p* > 0.05), log‐rank test. (b) Kaplan–Meier survival curves of ND‐fed female WT (*n* = 59) and ND‐fed female *Angptl2* ^−/−^ (*n* = 74) mice. ns, not significant (*p* > 0.05), log‐rank test. (c) Kaplan–Meier survival curves of HFD‐fed male WT (*n* = 58) and HFD‐fed male *Angptl2* ^−/−^ (*n* = 46) mice. ****p < 0.0001, log‐rank test. (d) Kaplan–Meier survival curves of HFD‐fed female WT (*n* = 66) and HFD‐fed female *Angptl2* ^−/−^ (*n* = 46) mice. ns, not significant (*p* > 0.05), log‐rank test. (e, f) Comparison of body weights over time in the indicated groups (ND: Up to 120 weeks; HFD: Up to 90 weeks). Data are means ± SE; *n* = 56, ND‐fed male WT; *n* = 113, ND‐fed male *Angptl2* ^−/−^; *n* = 68, HFD‐fed male WT; *n* = 42, HFD‐fed male *Angptl2* ^−/−^; *n* = 59, ND‐fed female WT; *n* = 74, ND‐fed female *Angptl2* ^−/−^; *n* = 66, HFD‐fed female WT; *n* = 45, HFD‐fed female *Angptl2* ^−/−^ mice. ****p < 0.0001; ***p < 0.001; **p < 0.01; *p < 0.05, two‐way ANOVA test.

To determine whether ANGPTL2 expression is associated with lifespan in mice, we generated *Angptl2*‐deficient (*Angptl2* ^−/−^) and wild‐type (WT) mice, fed them a normal chow diet (ND), and monitored their survival daily. Over the observation period, male *Angptl2* ^−/−^ mice exhibited a modest but not statistically significant reduction in survival compared to male WT mice (Figure 1a). The median survival was 124 weeks for *Angptl2* ^−/−^ mice and 125 weeks for WT mice. Analysis of postmortem findings (Table 1) revealed that lymphadenopathy was significantly less frequent in ND‐fed *Angptl2* ^−/−^ mice compared to WT mice (1 of 116 [9.5%] vs. 10 of 56 [17.9%]). Additionally, the incidence of tumors was significantly lower in ND‐fed *Angptl2* ^−/−^ mice (22 of 116 [19.0%] vs. 20 of 56 [35.7%]). Conversely, 12 of 116 (10.3%) ND‐fed *Angptl2* ^−/−^ mice developed intestinal hernias, a condition absent in ND‐fed WT mice. No other mortality factors showed significant differences between genotypes. We further investigated the impact of ANGPTL2 deficiency in female mice. Female *Angptl2* ^−/−^ mice showed a modest but not statistically significant reduction in survival compared to female WT mice under ND conditions (Figure 1b). The median survival was 117 weeks for *Angptl2* ^−/−^ mice and 124 weeks for WT mice.

**TABLE 1:** Postmortem findings (ND‐fed WT vs. ND‐fed *Angptl2* ^*−/−*^ mice).

	ND‐fed WT (*n* = 56)	ND‐fed *Angptl2* ^*−/−*^ (*n* = 116)	*p*
Cardiovascular			
Hypertrophy	1 (1.8%)	5 (4.3%)	0.6651
Lung			
Pleural fluid	1 (1.8%)	2 (1.7%)	1.000
Intestine			
Lymphadenopathy	10 (17.9%)	1 (9.5%)	< 0.0001
Hernia	0 (0.0%)	12 (10.3%)	0.0096
Rectal prolapse	0 (0.0%)	2 (1.7%)	1.000
Others	0 (0.0%)	4 (3.4%)	0.3051
Spleen			
Splenomegaly	3 (5.4%)	1 (9.5%)	0.1018
Genital			
Testicular enlargement	9 (16.0%)	8 (6.9%)	0.0984
Hernia	0 (0.0%)	5 (4.3%)	0.1748
Tumor			
Total	20 (35.7%)	22 (19.0%)	0.0187
Lung	4 (7.1%)	2 (1.7%)	0.0888
Intrapleural	1 (1.8%)	2 (1.7%)	1.000
Liver	13 (23.2%)	17 (14.7%)	0.1733
Intraperitoneal	2 (3.6%)	2 (1.7%)	0.5969

High‐fat diet (HFD)‐induced obesity is a known contributor to premature aging and mortality (Wagener et al. 2013). To examine the impact of ANGPTL2 deficiency on HFD‐induced premature aging, male *Angptl2* ^−/−^ and WT mice were fed a HFD starting at 6 weeks of age. Notably, HFD‐fed *Angptl2* ^−/−^ mice exhibited significantly increased mortality compared to HFD‐fed WT mice (Figure 1c). The median survival was 81.5 weeks for *Angptl2* ^−/−^ mice and 110 weeks for WT mice. Similar to the ND model, the incidence of intestinal hernia was significantly higher in HFD‐fed *Angptl2* ^−/−^ mice than in HFD‐fed WT mice (10 of 46 [21.7%] vs. 1 of 58 [1.7%]) (Table 2). Although lymphadenopathy, tumor formation, and testicular enlargement were observed less frequently in HFD‐fed *Angptl2* ^−/−^ mice compared to WT mice, these differences were not statistically significant. In addition, HFD‐fed female *Angptl2* ^−/−^ mice exhibited a modest but not statistically significant reduction in survival compared to HFD‐fed female WT mice (Figure 1d). The median survival was 98 weeks for *Angptl2* ^−/−^ mice and 107 weeks for WT mice. Next, we examined body weights of both WT and *Angptl2* ^−/−^ mice fed a ND or a HFD. Throughout the observation period, HFD‐fed male *Angptl2* ^−/−^ mice showed significantly lower body weight than HFD‐fed male WT mice (Figure 1e). Similarly, HFD‐fed female *Angptl2* ^−/−^ mice also exhibited significantly lower body weight than HFD‐fed female WT mice (Figure 1f). Notably, after 70 weeks of age in males and 80 weeks in females, HFD‐fed *Angptl2* ^−/−^ mice exhibited a progressive decline in body weight, suggesting systemic wasting or metabolic deterioration preceding death. These findings suggest that ANGPTL2 plays a crucial role in longevity in a mouse model of HFD‐induced premature aging in a potentially sex‐dependent manner.

**TABLE 2:** Postmortem findings (HFD‐fed WT vs. HFD‐fed *Angptl2* ^*−/−*^ mice).

	HFD‐fed WT (*n* = 58)	HFD‐fed *Angptl2* ^*−/−*^ (*n* = 46)	*p*
Cardiovascular			
Hypertrophy	5 (8.6%)	2 (4.3%)	0.4598
Ruptured aortic aneurysm	0 (0.0%)	1 (2.2%)	0.4423
Lung			
Chylothorax	4 (6.9%)	1 (2.2%)	0.3799
Intestine			
Lymphadenopathy	9 (15.5%)	2 (4.3%)	0.1066
Hernia	1 (1.7%)	10 (21.7%)	0.0021
Rectal prolapse	0 (0.0%)	1 (2.2%)	0.4423
Others	4 (6.9%)	2 (4.3%)	0.6913
Spleen			
Splenomegaly	1 (1.7%)	0 (0.0%)	1.000
Urinary			
Hydronephrosis	0 (0.0%)	2 (4.3%)	0.1932
Bladder dilatation	0 (0.0%)	2 (4.3%)	0.1932
Genital			
Testicular enlargement	10 (17.2%)	2 (4.3%)	0.0619
Hernia	1 (1.7%)	4 (8.7%)	0.1677
Tumor			
Total	29 (50.0%)	15 (32.6%)	0.0731
Cardiac	1 (1.7%)	0 (0.0%)	1.000
Lung	2 (3.4%)	2 (4.3%)	1.000
Intrapleural	0 (0.0%)	1 (2.2%)	0.4423
Liver	24 (41.4%)	13 (28.3%)	0.1629
Intestine	1 (1.7%)	0 (0.0%)	1.000
Intraperitoneal	1 (1.7%)	0 (0.0%)	1.000

### 2.2 ANGPTL2 Deficiency Attenuates High‐Fat Diet‐Induced Inflammation

**FIGURE 2:**
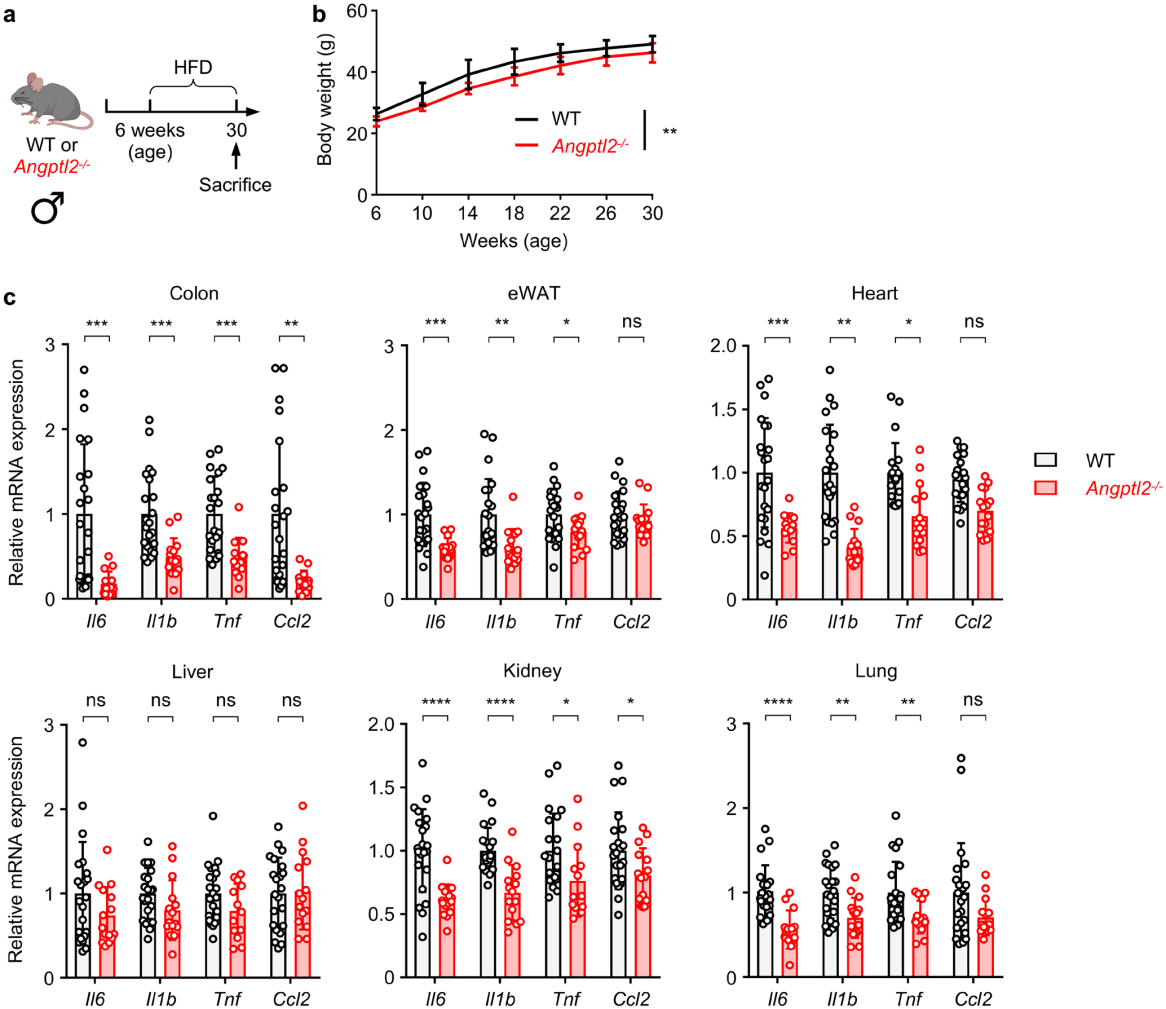
ANGPTL2 deficiency attenuates high‐fat diet‐induced systemic inflammation. (a) Schematic illustrating experimental design of the HFD‐induced premature aging model. (b) Comparison of body weights over time of HFD‐fed WT (*n* = 14) and HFD‐fed *Angptl2* ^−/−^ (*n* = 7) mice. Data are means ± SD. **p < 0.01, two‐way ANOVA test. (c) qRT‐PCR analysis of transcripts of indicated genes in colon, eWAT, heart, liver, kidney, and lung tissues from HFD‐fed WT (*n* = 22) and HFD‐fed *Angptl2* ^−/−^ (*n* = 15) mice at 30 weeks of age. WT levels were set to 1. Data are means ± SD. ns, not significant (*p* > 0.05); ****p < 0.0001; ***p < 0.001; **p < 0.01; *p < 0.05, unpaired *t*‐test, following outlier removal.

Since ANGPTL2 is a pro‐inflammatory mediator (Kadomatsu et al. 2014), we next assessed the expression of transcripts encoding pro‐inflammatory cytokines and chemokines in six major organs: colon, epididymal white adipose tissue (eWAT), heart, liver, lung, and kidney, of HFD‐fed *Angptl2* ^−/−^ and WT mice. Male mice were fed a HFD for 24 weeks starting at 6 weeks of age (Figure 2a). We performed tissue analyses at 30 weeks of age because significant mortality in HFD‐fed *Angptl2* ^−/−^ mice typically begins around this time point. This design allowed us to capture early pathological alterations preceding death. Over this period, *Angptl2* ^−/−^ mice exhibited significantly lower body weight than WT mice (Figure 2b). qRT‐PCR analysis revealed that the expression levels of *Il6*, *Il1b*, *Tnf*, and *Ccl2* were lower in five organs (colon, eWAT, heart, lung, and kidney) of HFD‐fed *Angptl2* ^−/−^ mice compared to WT mice (Figure 2c). Although liver samples from *Angptl2* ^−/−^ mice also showed reduced expression of these cytokines, the decrease was not statistically significant (Figure 2c). Overall, these results indicate that ANGPTL2‐mediated inflammation contributes to longevity regulation in a mouse model of HFD‐induced premature aging.

### 2.3 ANGPTL2 Is Important for Intestinal Regeneration During High‐Fat Diet Feeding

**FIGURE 3:**
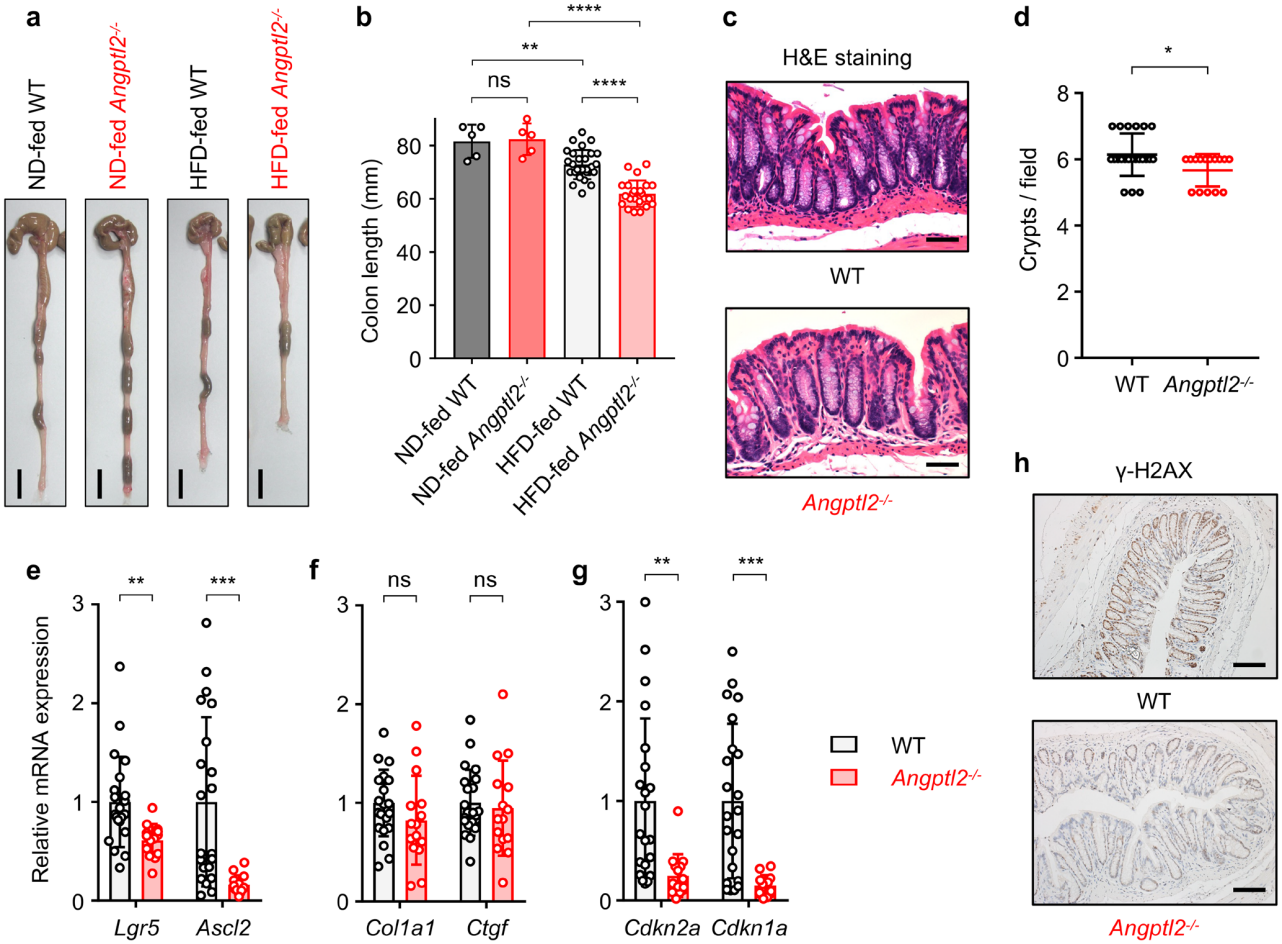
ANGPTL2 supports intestinal regeneration under metabolic stress. WT and *Angptl2* ^−/−^ mice were fed an HFD as seen in Figure 2a, starting at 6 weeks of age. (a, b) Representative images of colons (a) and colon length (b) from ND‐ or HFD‐fed WT and *Angptl2* ^−/−^ mice at 30 weeks of age. Scale bar, 1 cm. Data are means ± SD: *n* = 5, ND‐fed WT group; *n* = 5, ND‐fed *Angptl2* ^−/−^ group; *n* = 27, HFD‐fed WT group; *n* = 23, HFD‐fed *Angptl2* ^−/−^ group. ns, not significant (*p* > 0.05); ****p < 0.0001; **p < 0.01, one‐way ANOVA test followed by Tukey's multiple comparison test. (c) Representative images of H&E‐stained colon tissue from HFD‐fed WT and HFD‐fed *Angptl2* ^−/−^ mice at 30 weeks of age. Scale bar, 50 μm. (d) The number of crypts as quantified using H&E‐stained sections shown in (c). Each data point represents the average number of crypts in a single field of view. Multiple areas in section per mouse were quantified. Data are means ± SD. *p < 0.05, unpaired *t*‐test. (e–g) qRT‐PCR analysis of transcripts of indicated genes in colon tissues from HFD‐fed WT (*n* = 22) and HFD‐fed *Angptl2* ^−/−^ (*n* = 15) mice at 30 weeks of age. WT levels were set to 1. Data are means ± SD. ns, not significant (*p* > 0.05); ***p < 0.001; **p < 0.01, unpaired *t*‐test, following outlier removal. (h) Representative images of γ‐H2AX immunostaining in colon tissue from HFD‐fed WT and HFD‐fed *Angptl2* ^−/−^ mice at 30 weeks of age. Scale bar, 200 μm.

Given that HFD feeding disrupts the intestinal barrier (Rohr et al. 2020; Yang et al. 2025) and that intestinal hernias were observed as a postmortem finding in *Angptl2* ^−/−^ mice, we explored the role of ANGPTL2 in the colon. In our HFD model, WT mice fed a HFD exhibited a significant reduction in colon length compared to ND‐fed WT mice. Interestingly, HFD‐fed *Angptl2* ^−/−^ mice displayed marked shortening of the colon compared to HFD‐fed WT mice, indicative of severe tissue injury (Figure 3a,b). Histological analysis revealed a significant reduction in the number of crypts in the colons of HFD‐fed *Angptl2* ^−/−^ mice (Figure 3c,d). Because HFD enhances intestinal stemness (Beyaz et al. 2016), we examined the expression of intestinal stem cell (ISC) markers *Lgr5* and *Ascl2*. qRT‐PCR analysis demonstrated significantly reduced expression of these markers in the colons of HFD‐fed *Angptl2* ^−/−^ mice compared to HFD‐fed WT mice (Figure 3e), suggesting that ANGPTL2 is required for ISC maintenance and effective intestinal regeneration following HFD‐induced damage. On the other hand, expression levels of fibrosis markers *Col1a1* and *Ctgf* in the colon were comparable between HFD‐fed WT and *Angptl2* ^−/−^ mice (Figure 3f). Recent studies have suggested that cellular senescence plays a crucial physiological role in tissue regeneration (Yao et al. 2023; Cheng et al. 2022). qRT‐PCR analysis showed that *Cdkn2a* (p16) and *Cdkn1a* (p21), both markers of cellular senescence, were significantly downregulated in the colons of HFD‐fed *Angptl2* ^−/−^ mice compared to HFD‐fed WT mice (Figure 3g). Furthermore, γ‐H2AX, a sensitive marker of DNA damage and associated with cellular senescence, was predominantly detected in the nuclei of crypt epithelial cells in WT mice relative to *Angptl2* ^−/−^ mice (Figure 3h), supporting the idea of reduced cellular senescence in the absence of ANGPTL2. These findings suggest that ANGPTL2‐mediated inflammation and cellular senescence contribute to effective intestinal regeneration following HFD‐induced damage.

### 2.4 ANGPTL2 Deficiency Attenuates Pathological Tissue Remodeling Induced by a High‐Fat Diet

**FIGURE 4:**
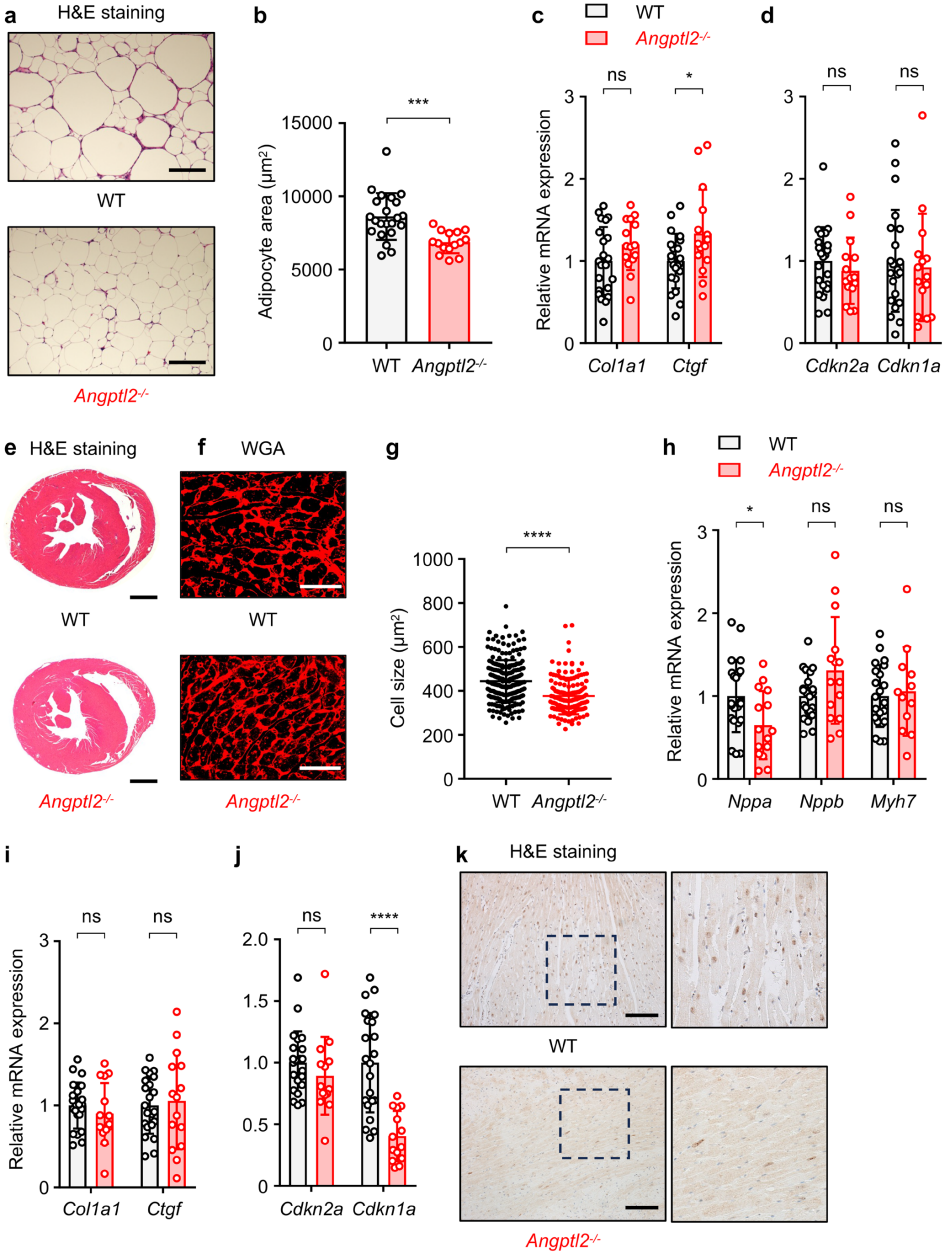
ANGPTL2 promotes pathological tissue remodeling in adipose tissue and heart. WT and *Angptl2* ^−/−^ mice were fed an HFD as seen in Figure 2a, starting at 6 weeks of age. (a, b) Representative images of H&E‐stained eWAT (a) and adipocyte area (b) from HFD‐fed WT and HFD‐fed *Angptl2* ^−/−^ mice at 30 weeks of age. Scale bar, 250 μm. Data are means ± SD: *n* = 22, HFD‐fed WT group; *n* = 15, HFD‐fed *Angptl2* ^−/−^ group. ***p < 0.001, unpaired *t*‐test. (c, d) qRT‐PCR analysis of transcripts of indicated genes in eWAT from HFD‐fed WT (*n* = 22) and HFD‐fed *Angptl2* ^−/−^ (*n* = 15) mice at 30 weeks of age. WT levels were set to 1. Data are means ± SD. ns, not significant (*p* > 0.05); *p < 0.05, unpaired *t*‐test. (e) Representative images of H&E‐stained heart tissues from HFD‐fed WT and HFD‐fed *Angptl2* ^−/−^ mice at 30 weeks of age. Scale bar, 1 mm. (f, g) Representative images of WGA‐stained heart tissues (f) and cardiomyocyte size (g) from HFD‐fed WT and HFD‐fed *Angptl2* ^−/−^ mice at 30 weeks of age. Scale bar, 50 μm. Data are means ± SD: *n* = 22, HFD‐fed WT group; *n* = 15, HFD‐fed *Angptl2* ^−/−^ group. ****p < 0.0001, unpaired *t*‐test. (h–j) qRT‐PCR analysis of transcripts of indicated genes in heart tissues from HFD‐fed WT (*n* = 22) and HFD‐fed *Angptl2* ^−/−^ (*n* = 15) mice at 30 weeks of age. WT levels were set to 1. Data are means ± SD. ns, not significant (*p* > 0.05); ****p < 0.0001; *p < 0.05, unpaired *t*‐test, following outlier removal. (k) Representative images of γ‐H2AX immunostaining in heart tissue from HFD‐fed WT and HFD‐fed *Angptl2* ^−/−^ mice at 30 weeks of age. Scale bar, 200 μm. Right panel shows magnification of the corresponding square in left panel.

Because we previously reported that deletion of ANGPTL2 reduces adipose tissue inflammation and systemic insulin resistance in dietary obesity (Tabata et al. 2009), we further investigated a possible role of ANGPTL2 in HFD‐induced adipose tissue remodeling. Histological analysis revealed that adipocyte size was significantly reduced in HFD‐fed *Angptl2* ^−/−^ mice compared to HFD‐fed WT mice in our HFD model (Figure 4a,b), suggesting decreased adipocyte hypertrophy. However, qRT‐PCR analysis showed that *Col1a1* expression was not significantly different between genotypes, and *Ctgf* expression was only minimally affected (Figure 4c). Additionally, *Cdkn2a* and *Cdkn1a* expression levels were comparable between genotypes (Figure 4d). These results indicate that while ANGPTL2‐mediated inflammation promotes adipocyte hypertrophy but does not significantly contribute to adipose tissue fibrosis or cellular senescence.

HFD‐induced obesity is a major contributor to cardiac dysfunction and remodeling (Zeng et al. 2015). To investigate a possible role of ANGPTL2 in HFD‐induced cardiac dysfunction, we performed histological analyses of heart tissue. Hematoxylin and eosin (H&E) staining and wheat germ agglutinin (WGA) staining revealed significantly reduced cardiomyocyte size in HFD‐fed *Angptl2* ^−/−^ mice compared to HFD‐fed WT mice (Figure 4e–g), indicating attenuated cardiomyocyte hypertrophy. Furthermore, qRT‐PCR analysis showed significantly lower transcript levels of *Nppa*, a marker of heart failure, in the hearts of HFD‐fed *Angptl2* ^−/−^ mice compared to HFD‐fed WT mice; however, expression of other heart failure markers (*Nppb* and *Myh7*) and fibrosis markers (*Col1a1* and *Ctgf*) were comparable between genotypes (Figure 4h,i). Notably, *Cdkn1a* expression was significantly lower in the hearts of HFD‐fed *Angptl2* ^−/−^ mice, while *Cdkn2a* showed a modest but not significant decrease (Figure 4j). Furthermore, γ‐H2AX was detected in cardiomyocytes of WT mice, whereas *Angptl2* ^−/−^ mice exhibited fewer γ‐H2AX‐positive cardiomyocytes (Figure 4k), suggesting attenuated cellular senescence‐associated DNA damage in the absence of ANGPTL2. Given that cardiac senescence is a potential driver of cardiovascular disease (Luan et al. 2024), these findings suggest that ANGPTL2‐mediated inflammation and cellular senescence contribute to HFD‐induced heart failure.

### 2.5 ANGPTL2 Has Organ‐Dependent Opposing Effects on Tissue Fibrosis

**FIGURE 5:**
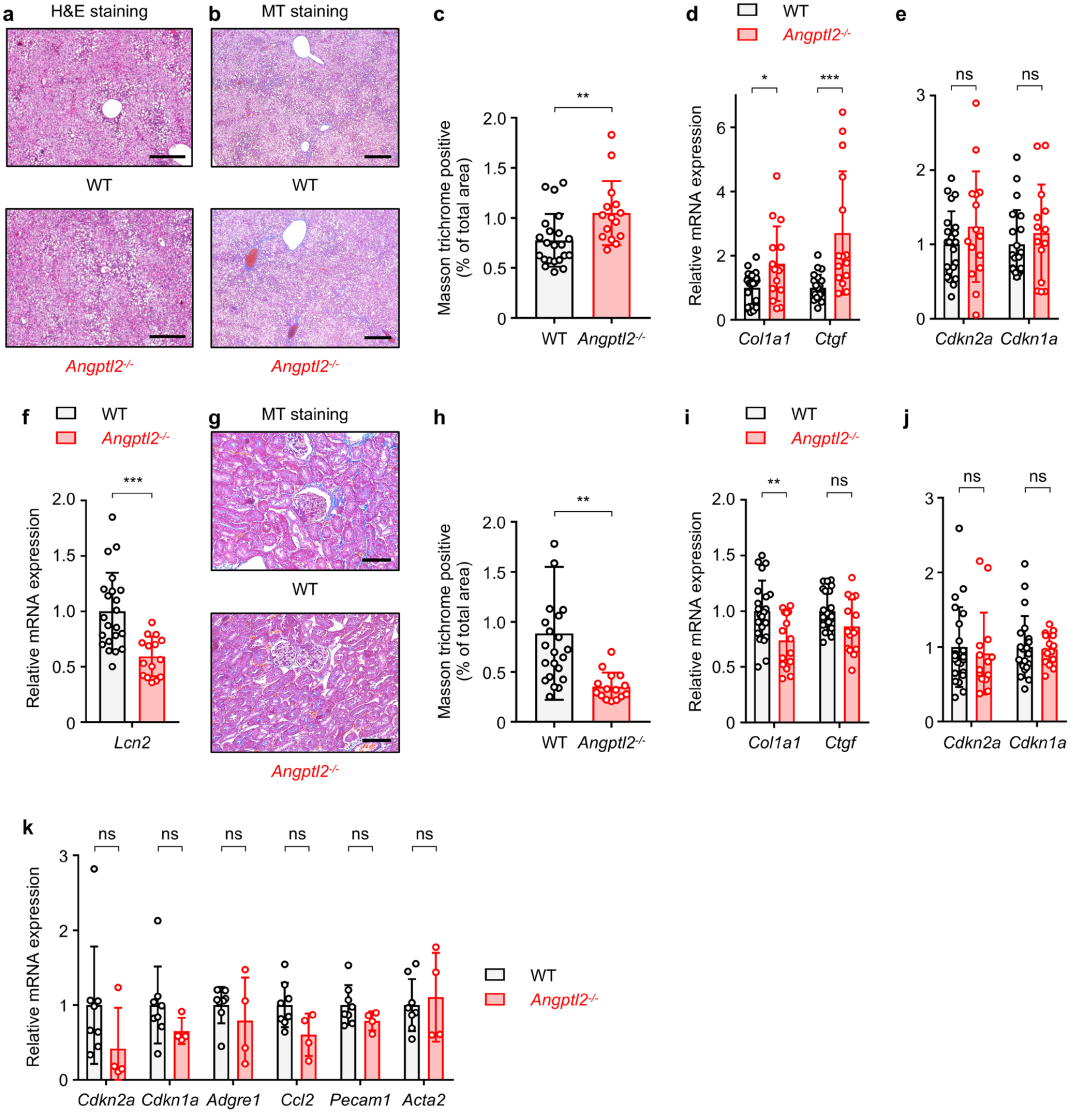
Organ‐specific roles of ANGPTL2 in regulating tissue fibrosis. WT and *Angptl2* ^−/−^ mice were fed an HFD as seen in Figure 2a, starting at 6 weeks of age. (a) Representative images of H&E‐stained liver tissue from HFD‐fed WT and HFD‐fed *Angptl2* ^−/−^ mice at 30 weeks of age. Scale bar, 500 μm. (b, c) Representative images of Masson's trichrome‐stained liver tissues (b) and fibrotic areas (c) from HFD‐fed WT and HFD‐fed *Angptl2* ^−/−^ mice at 30 weeks of age. Scale bar, 500 μm. Data are means ± SD: *n* = 22, HFD‐fed WT group; *n* = 15, HFD‐fed *Angptl2* ^−/−^ group. **p < 0.01, unpaired *t*‐test. (d, e) qRT‐PCR analysis of transcripts of indicated genes in liver tissues from HFD‐fed WT (*n* = 22) and HFD‐fed *Angptl2* ^−/−^ (*n* = 15) mice at 30 weeks of age. WT levels were set to 1. Data are means ± SD. ns, not significant (*p* > 0.05); ***p < 0.001; *p < 0.05, unpaired *t*‐test, following outlier removal. (f) qRT‐PCR analysis of *Lcn2* transcripts in kidney tissues from HFD‐fed WT (*n* = 22) and HFD‐fed *Angptl2* ^−/−^ (*n* = 15) mice at 30 weeks of age. WT levels were set to 1. Data are means ± SD. ***p < 0.001; *p < 0.05, unpaired *t*‐test. (g, h) Representative images of Masson's trichrome‐stained kidney tissues (g) and fibrotic areas (h) from HFD‐fed WT and HFD‐fed *Angptl2* ^−/−^ mice at 30 weeks of age. Scale bar, 50 μm. Data are means ± SD: *n* = 22, HFD‐fed WT group; *n* = 15, HFD‐fed *Angptl2* ^−/−^ group. **p < 0.01, unpaired *t*‐test. (i, j) qRT‐PCR analysis of transcripts of indicated genes in kidney tissues from HFD‐fed WT (*n* = 22) and HFD‐fed *Angptl2* ^−/−^ (*n* = 15) mice at 30 weeks of age. WT levels were set to 1. Data are means ± SD. ns, not significant (*p* > 0.05); **p < 0.01, unpaired *t*‐test, following outlier removal. (k) qRT‐PCR analysis of transcripts of indicated genes in PRAT from HFD‐fed WT (*n* = 8) and HFD‐fed *Angptl2* ^−/−^ (*n* = 4) mice at 30 weeks of age. WT levels were set to 1. Data are means ± SD. ns, not significant (*p* > 0.05), unpaired *t* test.

Histological examination of liver tissue from HFD‐fed WT and *Angptl2* ^−/−^ mice revealed no significant differences in hepatic steatosis or hepatocellular ballooning, as assessed by H&E staining (Figure 5a). However, Masson's trichrome (MT) staining indicated that accumulation of perivascular collagen fibers was observed in livers from HFD‐fed WT mice, whereas perivascular fibrosis was significantly increased in livers from HFD‐fed *Angptl2* ^−/−^ mice relative to HFD‐fed WT (Figure 5b,c). Moreover, qRT‐PCR analysis showed that hepatic expression of *Col1a1* and *Ctgf* was significantly higher in HFD‐fed *Angptl2* ^−/−^ mice compared with HFD‐fed WT mice (Figure 5d). On the other hand, levels of *Cdkn2a* and *Cdkn1a*, markers of cellular senescence, were comparable between genotypes (Figure 5e). Given that expression levels of pro‐inflammatory cytokines were not significantly different between genotypes (Figure 2c), these findings suggest that ANGPTL2 protects against HFD‐induced perivascular fibrosis in the liver through a mechanism independent of inflammation and cellular senescence.

Since HFD promotes renal injury (Sun et al. 2020), we next examined the role of ANGPTL2 in HFD‐induced kidney injury and fibrosis. Expression of *Lcn2*, a marker of kidney injury encoding neutrophil gelatinase‐associated lipocalin (NGAL), was significantly lower in kidneys from HFD‐fed *Angptl2* ^−/−^ mice relative to HFD‐fed WT mice (Figure 5f), suggesting that ANGPTL2 contributes to HFD‐induced renal injury. Furthermore, MT staining and qRT‐PCR analysis demonstrated that ANGPTL2 deficiency prevents kidney fibrosis (Figure 5g–i). However, expression levels of *Cdkn2a* and *Cdkn1a* were comparable between genotypes, indicating that ANGPTL2 deficiency did not significantly affect renal cellular senescence (Figure 5j). These findings indicate that ANGPTL2 plays a dual role in tissue fibrosis, protecting against perivascular fibrosis in the liver while exacerbating kidney injury and fibrosis in the context of HFD‐induced metabolic stress.

Given that perirenal adipose tissue (PRAT) can influence kidney function (Fazeli et al. 2024), to better understand the protective role of ANGPTL2 deficiency against HFD‐induced renal fibrosis, we examined a potential role of ANGPTL2 in PRAT. qRT‐PCR analysis revealed lower expression levels of *Cdkn2a* and *Cdkn1a* transcripts in PRAT from *Angptl2* ^−/−^ mice compared with WT mice, although the changes were not statistically significant (Figure 5k). We also observed modest but not significant decreases in *Ccl2* expression in *Angptl2* ^−/−^ PRAT; however, the expression of the macrophage/monocyte marker *Adgre1* (F4/80) was comparable between genotypes (Figure 5k). In addition, there were no significant differences in the expression of the endothelial marker *Pecam1* or the vascular smooth muscle marker *Acta2* between WT and *Angptl2* ^−/−^ mice (Figure 5k). Collectively, these results suggest that ANGPTL2 does not directly affect immune cell infiltration or vascular remodeling in PRAT, but rather that ANGPTL2 expression within the kidney contributes to HFD‐induced renal injury and fibrosis.

### 2.6 ANGPTL2 Promotes High‐Fat Diet‐Induced Lymphocyte Aggregation in the Lung

**FIGURE 6:**
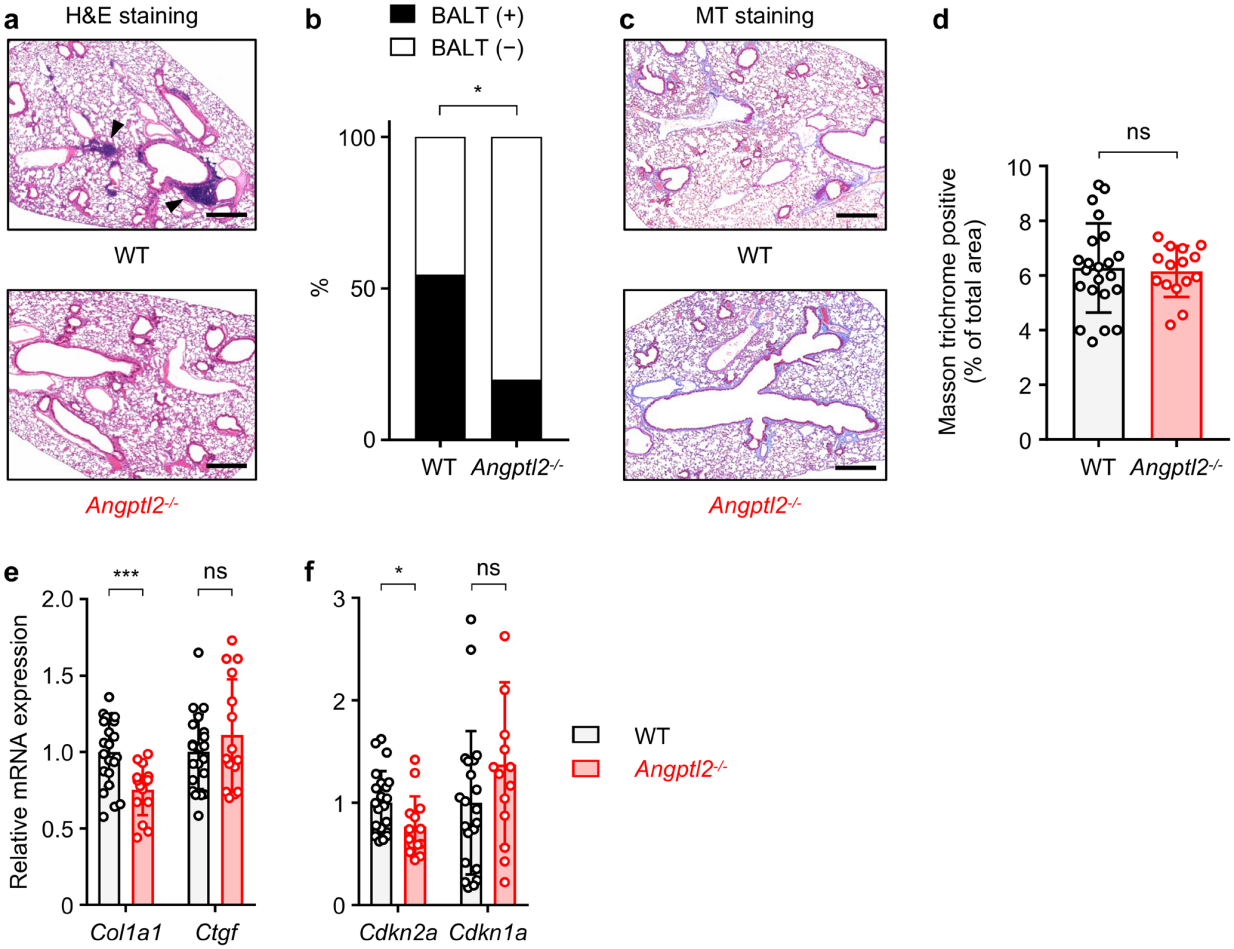
ANGPTL2 promotes lymphocyte aggregation in the lung. WT and *Angptl2* ^−/−^ mice were fed an HFD as seen in Figure 2a, starting at 6 weeks of age. (a) Representative images of H&E‐stained lung tissue from HFD‐fed WT and HFD‐fed *Angptl2* ^−/−^ mice at 30 weeks of age. Scale bar, 500 μm. Arrowheads indicate a BALT. (b) Proportion of lung tissues having BALT shown in (a). *n* = 22, HFD‐fed WT group; *n* = 15, HFD‐fed *Angptl2* ^−/−^ group. *p < 0.05, Fisher's exact test. (c, d) Representative images of Masson's trichrome‐stained lung tissues (c) and fibrotic areas (d) from HFD‐fed WT and HFD‐fed *Angptl2* ^−/−^ mice at 30 weeks of age. Scale bar, 500 μm. Data are means ± SD: *n* = 22, HFD‐fed WT group; *n* = 15, HFD‐fed *Angptl2* ^−/−^ group. **p < 0.01, unpaired *t*‐test. (e, f) qRT‐PCR analysis of transcripts of indicated genes in lung tissues from HFD‐fed WT (*n* = 22) and HFD‐fed *Angptl2* ^−/−^ (*n* = 15) mice at 30 weeks of age. WT levels were set to 1. Data are means ± SD. ns, not significant (*p* > 0.05); ***p < 0.001; *p < 0.05, unpaired *t*‐test, following outlier removal.

To further assess the role of ANGPTL2 in HFD‐induced tissue remodeling, we performed histopathological analysis of lung tissue from HFD‐fed WT and HFD‐fed *Angptl2* ^−/−^ mice. Lymphocyte aggregation, resembling bronchus‐associated lymphoid tissues (BALTs), was observed in peribronchial and perivascular regions of the lungs from HFD‐fed WT mice (Figure 6a). BALTs were detected in 12 of 20 (60%) HFD‐fed WT mice but only in 3 of 15 (20%) HFD‐fed *Angptl2* ^−/−^ mice, indicating a significant reduction in BALT formation in the absence of ANGPTL2 (Figure 6a,b). However, pulmonary fibrosis, as assessed by MT staining, was comparable between genotypes (Figure 6c,d), although *Col1a1* expression was lower in lungs from HFD‐fed *Angptl2* ^−/−^ mice (Figure 6e). In addition, qRT‐PCR analysis revealed that transcript levels of *Cdkn2a* were only minimally decreased in lungs from HFD‐fed *Angptl2* ^−/−^ mice compared with HFD‐fed WT mice (Figure 6f). These findings suggest that ANGPTL2 promotes HFD‐induced BALT formation in the lung. Given that the functional significance of BALT formation is context‐dependent (Foo and Phipps 2010), further investigation is required to determine whether ANGPTL2‐mediated BALT formation is beneficial or detrimental in the setting of HFD‐induced metabolic stress.

## 3 Discussion

**FIGURE 7:**
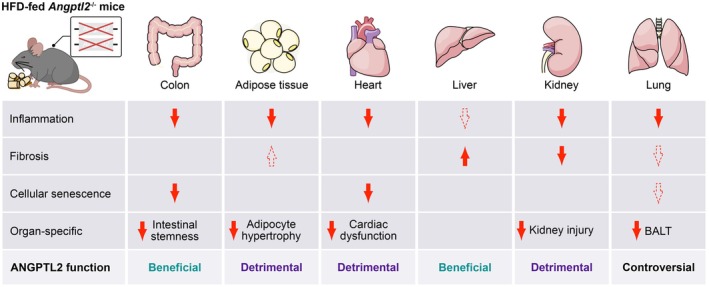
Schematic summary of organ‐specific effects of ANGPTL2 deficiency in high‐fat diet‐fed mice. The impact of ANGPTL2 deficiency on various organs including the colon, adipose tissue, heart, liver, kidney, and lung. The overall functional role of ANGPTL2 in each organ is classified as beneficial, detrimental, or controversial.

Here, we demonstrate the dual role of ANGPTL2 in regulating inflammation, tissue regeneration, and disease progression in the context of HFD‐induced pathology. ANGPTL2 promotes pro‐inflammatory responses, contributing to local inflammation, adipocyte hypertrophy, and cardiac dysfunction. Conversely, ANGPTL2 exerts protective effects by supporting intestinal regeneration and vascular integrity in the liver, highlighting its essential role in maintaining tissue homeostasis. Additionally, ANGPTL2 is involved in the formation of BALTs in the lung, suggesting a complex role in immune regulation with potential context‐dependent implications for pulmonary health. These findings indicate the need to consider the organ‐ and context‐specific functions of ANGPTL2 (Figure 7).

Aging and obesity are both associated with the accumulation of senescent cells, which secrete SASP factors, including pro‐inflammatory cytokines, chemokines, and extracellular matrix remodeling proteins. These chronic inflammations play a central role in the pathogenesis of various age‐related diseases, including metabolic disorders, cardiovascular disease, and fibrosis (Minamino et al. 2009; Ajoolabady et al. 2023; Murtha et al. 2019). Given that ANGPTL2 expression is upregulated in aging and obesity, particularly in adipocytes and macrophages within adipose tissue (Horio et al. 2014; Tabata et al. 2009), its role as a SASP factor is particularly relevant in obesity‐induced metabolic inflammation. Beyond adipose tissue, ANGPTL2 may also function as a SASP factor in other senescent cell populations, such as vascular endothelial cells and fibroblasts. Excessive activation of ANGPTL2 signaling in these cells could drive endothelial dysfunction, tissue fibrosis, and organ degeneration, hallmarks of inflammaging‐related pathology. The present study supports this hypothesis, as ANGPTL2 deficiency reduced inflammation across multiple organs and attenuated obesity‐induced cardiac dysfunction, kidney fibrosis, and adipocyte hypertrophy. However, our data also indicate that ANGPTL2 plays a protective role in certain contexts, such as maintaining intestinal homeostasis and preventing perivascular fibrosis in the liver. This dual role of ANGPTL2 suggests the importance of context‐dependent regulation of SASP factors.

In this study, ANGPTL2 deficiency had a clear impact on lifespan in male mice, whereas the effect was less pronounced and not statistically significant in females. Previous studies have demonstrated that senescence pathways can be activated in a sex‐dependent manner, as evidenced by differential responses to senolytic interventions in rodents and humans (Fang et al. 2023; Schwab et al. 2022; Budamagunta et al. 2023; Xia et al. 2024; Mury et al. 2025). Therefore, it is possible that ANGPTL2 deficiency has distinct effects on senescence‐associated phenotypes in males and females. Future studies are needed to address this point to fully elucidate the role of ANGPTL2 in sex‐specific regulation of tissue homeostasis and aging.

HFD induces metabolic stress and intestinal barrier dysfunction (Rohr et al. 2020). Since the intestinal lumen is directly exposed to dietary stress, the intestinal epithelium is characterized by rapid and continuous renewal throughout an animal's life to re‐establish the epithelial barrier after mucosal injury. In our study, ANGPTL2‐deficient mice exhibited a shortened lifespan under HFD‐fed conditions. Furthermore, ANGPTL2 deficiency exacerbated HFD‐induced intestinal damage, as demonstrated by increased colon shortening, reduced crypt numbers, and decreased expression of ISC markers. These findings suggest that ANGPTL2‐mediated inflammatory responses may regulate intestinal homeostasis during HFD‐induced injury. Indeed, previous studies demonstrated that *angptl2* expression is induced during fin regeneration in zebrafish, particularly in blastema tissue, suggesting a role for ANGPTL2 in tissue repair (Kubota et al. 2005). Notably, we previously reported that ANGPTL2 promotes intestinal epithelial regeneration following dextran sulfate sodium (DSS)‐induced injury (Horiguchi et al. 2017). Therefore, in the absence of ANGPTL2, impaired epithelial regeneration disrupts gut barrier integrity, thereby leading to translocation of microbial components into the body and the reduced survival observed in ANGPTL2‐deficient mice under HFD‐fed conditions.

Tissue fibrosis is a pathological process characterized by excessive extracellular matrix deposition, leading to organ dysfunction. ANGPTL2 is reportedly implicated in fibrosis across multiple organs (Morinaga et al. 2016; Motokawa et al. 2016; Tian et al. 2016), but its effects are organ‐specific, exhibiting both pro‐fibrotic and anti‐fibrotic properties depending on the tissue context. In the present study, we found that ANGPTL2 deficiency attenuated kidney fibrosis and reduced the expression of fibrosis‐related genes, suggesting that ANGPTL2 promotes fibrotic remodeling in the kidney under HFD‐fed conditions, consistent with previous reports demonstrating that ANGPTL2 exacerbates renal fibrosis in a mouse unilateral ureteral obstruction model (Morinaga et al. 2016). Similarly, ANGPTL2 has been shown to contribute to cardiac fibrosis in response to pressure overload‐induced stress (Tian et al. 2016), supporting its role as a pro‐fibrotic factor. In contrast, our data indicate that ANGPTL2 plays a protective role against perivascular fibrosis in the liver. Furthermore, we previously reported that ANGPTL2 deficiency exacerbates bleomycin‐induced fibrosing interstitial pneumonia (Motokawa et al. 2016), supporting its role as an anti‐fibrotic factor. These findings suggest the complex and organ‐dependent roles of ANGPTL2 in fibrosis. Further studies are needed to elucidate the molecular pathways that determine its pro‐ or anti‐fibrotic roles in different tissue environments.

Our study also demonstrates that ANGPTL2 promotes lymphoid tissue remodeling, including lymph node hypertrophy and HFD‐induced BALT formation. The presence of BALT is generally associated with enhanced immune responses (Moyron‐Quiroz et al. 2004); however, excessive BALT formation has been implicated in autoimmunity and chronic lung diseases (Hogg et al. 2004; Foo and Phipps 2010). ANGPTL2‐mediated inflammatory chemokine expression may facilitate immune cell recruitment to the lung, potentially driving BALT formation in chronic inflammatory conditions. Together, these findings suggest that ANGPTL2 is a crucial regulator of immune responses, promoting lymphoid tissue remodeling, lymph node enlargement, and BALT formation. These effects may enhance chronic inflammation, host defense, and even antitumor immunity.

Postmortem analysis of HFD‐fed ANGPTL2‐deficient mice revealed reduced tumor development, which is consistent with our previous findings that ANGPTL2 signaling in tumor cells promotes tumor progression (Horiguchi et al. 2014, 2022). Notably, we recently reported that obesity‐induced chronic inflammation, facilitated by high ANGPTL2 expression, suppresses the efficacy of ICIs (Yumoto et al. 2024). Nonetheless, we recently reported that ANGPTL2 facilitates CD8^+^ T cell cross‐priming and enhances antitumor immune responses (Horiguchi et al. 2019, 2021; Horiguchi, Kadomatsu, Yamashita, et al. 2024). Moreover, ANGPTL2 has also been shown to promote immune checkpoint inhibitor (ICI)‐related immune‐related adverse events (irAEs) (Horiguchi et al. 2023). A possible explanation for these contradictory effects is that ANGPTL2 plays distinct roles depending on its source: tumor cell‐derived ANGPTL2 promotes tumor progression, whereas stromal cell‐derived ANGPTL2 may have tumor‐suppressive effects (Horiguchi, Kadomatsu, and Oike 2024). Further studies are needed to dissect these mechanisms and clarify the conditions under which ANGPTL2 acts as a tumor promoter versus a tumor suppressor.

A recent report demonstrated that *Angptl2* knockdown leads to mild cardiac valve malformations due to impaired programmed senescence during development (Labbé et al. 2022). Although we did not observe any overt developmental defects in ANGPTL2‐deficient mice, we cannot exclude the possibility of subtle embryonic abnormalities that may not be detected in our study. Therefore, developmental alterations associated with impaired cellular senescence might predispose individuals to impaired tissue repair later in life, and that such latent susceptibility could have been unmasked by HFD‐induced stress in our model.

In summary, our findings highlight the dual roles of ANGPTL2 in inflammation, tissue homeostasis, and immune regulation, indicating its context‐dependent functions in aging, obesity, and chronic disease. Our findings, using ANGPTL2 as a proxy for cellular senescence, further indicate the crucial role of this pathway as a defense and repair mechanism even in adult and aged animals. The impact of cellular senescence appears to be heterogeneous, depending on the cell type and likely influenced by the age and sex of the organism. Moreover, cellular senescence‐associated pathways may be more detrimental in low‐regenerating organs such as the heart and kidneys, while protective in tissues with high cellular turnover, including the intestinal epithelium, liver, and immune system. This finding suggests that the maintenance of healthspan may depend on the integrity of high‐turnover systems. Therefore, designing therapeutic interventions will require careful consideration of the complex and opposing effects of ANGPTL2 in different cellular and organ contexts.

## 4 Materials and Methods

### 4.1 Animals

All experimental procedures were approved by the Ethics Review Committee for Animal Experimentation of Kumamoto University (A21‐278, A22‐056, A23‐123, A24‐060, A25‐184, and A2023‐087). *Angptl2* ^+/−^ mice (Tabata et al. 2009) were backcrossed to a C57BL/6N strain for at least ten generations. All animals were housed in a controlled environment with an automated 12‐h light/dark cycle and a stable room temperature of 23°C and maintained under specific pathogen‐free (SPF) conditions. Mice showing clear signs of abnormal sickness unrelated to experimental conditions were excluded from the study. For lifespan analysis (Figure 1), both male and female mice were used. All other experiments (Figures 2, 3, 4, 5, 6) were performed using male mice only.

For lifespan analysis, mice were generated via in vitro fertilization (IVF) using sperm collected from littermate *Angptl2* ^+/+^ and *Angptl2* ^−/−^ mice. At 6 weeks of age, mice were randomly assigned to either a ND (CE‐2, CLEA) or a HFD (HFD‐32, CLEA). Mortality was monitored daily. Postmortem examination was performed by two independent researchers.

### 4.2 Total RNA Extraction and Real‐Time Quantitative RT‐PCR

Total RNA was isolated from tissues using TRI Reagent (Molecular Research Center). DNase‐treated RNA was reversed‐transcribed with a PrimeScript RT regent Kit (Takara Bio). PCR products were analyzed using a Thermal Cycler Dice Real Time System (Takara Bio). PCR primer sequences are provided in Table S1. Relative transcript abundance was normalized to that of *18s* mRNA.

### 4.3 Histology

For histological analysis, tissue samples were dissected, washed in ice‐cold PBS, and fixed in 15% neutral buffered formalin at room temperature for 24 h. Fixed tissues were embedded in paraffin, and 4‐μm sections were prepared following standard histological protocols. Routine H&E staining was performed for general histology. Cardiomyocyte size was quantified using Alexa Fluor 594‐conjugated WGA (Life Technologies) and DAPI staining. Tissue fibrosis was evaluated using Masson's trichrome staining, with fibrotic areas quantified by measuring blue‐stained regions. Adipocyte area, cardiomyocyte size, and fibrotic area were calculated using the BZ‐X analyzer version 1.4.1.1 (Keyence). For immunohistochemistry, after antigen retrieval, endogenous peroxidase was blocked using 3% H_2_O_2_ for 10 min. Samples were then blocked with 5% serum for 20 min at room temperature and incubated with anti‐gamma H2AX (phospho S139) (1:500, Abcam, ab11174) for 120 min at room temperature. Appropriate secondary antibodies were applied for 60 min at room temperature, and 0.02% DAB solution was used for detection and visualization of staining. Slides were counterstained with hematoxylin and mounted.

### 4.4 Statistical Analysis

Statistical analyses were performed using GraphPad prism 7 software (GraphPad Software). Statistical parameters and methods are reported in respective figures and figure legends. Results with *p* values < 0.05 were considered significant (*p < 0.05; **p < 0.01; ***p < 0.001; ****p < 0.0001). Comparisons between two groups were performed using an unpaired two‐tailed *t*‐test. Comparisons between three or more groups were performed using one‐way ANOVA with Tukey's multiple comparison test. Survival rate was analyzed by log‐rank test. For comparisons of body weight over time, we used a two‐way ANOVA. For the association of binary variables, we used Fisher's exact test. To determine statistical outliers, we used the Robust Regression and Outlier Removal (ROUT) method (Q = 1%) prior to data analysis.

## Author Contributions

S.Y. and H.H. designed the study, performed and analyzed experiments, and wrote the paper. K.M. and Z.T. performed lifespan analysis. T.K. and M.S. performed and analyzed experiments. K.A. provided *Angptl2* mutant mice. M.I. supervised the study. Y.O. coordinated, designed, and supervised the study. All authors discussed the data and commented on the paper.

## Funding

This work was supported by the Japan Agency for Medical Research and Development (18gm0610007h0006) and Ministry of Education, Culture, Sports, Science and Technology (23K06638).

## Conflicts of Interest

The authors declare no conflicts of interest.

## Supporting information


**Table S1:** Sequences of primers used in Real‐time PCR analysis.

## Data Availability

Data sharing not applicable to this article as no datasets were generated or analysed during the current study.
