# Supplementary material for: Context‐Dependent Roles of ANGPTL2‐Mediated Inflammaging in Tissue Homeostasis, Pathological Tissue Remodeling, and Longevity
Source: Aging Cell. 2026 Jan 8;25(2):e70370. doi: 10.1111/acel.70370 (PMC12783698; doi:10.1111/acel.70370)
Supplement: Supplementary file 1 — Table S1: Sequences of primers used in Real‐time PCR analysis. [file ACEL-25-e70370-s001.docx]

**Supplemental Table 1. Sequences of primers used in Real-time PCR analysis**

Gene Forward primer Reverse primer

*Il6* AAGTCGGAGGCTTAATTACACATGT CCATTGCACAACTCTTTTCTCATTC

*Il1b* TCCAGGATGAGGACATGAGCAC GAACGTCACACACCAGCAGGTT

*Tnf* AAGCCTGTAGCCCACGTCGTA GGCACCACTAGTTGGTTGTCTTTG

*Ccl2* CATCCACGTGTTGGCTCA GATCATCTTGCTGGTGAATGAGT

*Lgr5* TGCCCCGTGGCTTTCTTATC TTTCCCAGGCTGCCCATATC

*Ascl2* AAGCACACCTTGACTGGTACG AAGTGGACGTTTGCACCTTCA

*Col1a1* GACATGTTCAGCTTTGTGGACCTC GGGACCCTTAGGCCATTGTGTA

*Ctgf* CAAAGCAGCTGCAAATACCA GGCCAAATGTGTCTTCCAGT

*Cdkn2a* GTGTGCATGACGTGCGGG GCAGTTCGAATCTGCACCGTAG

*Cdkn1a* GCCTTAGCCCTCACTCTGTG AGCTGGCCTTAGAGGTGACA

*Nppa* GAGAGACGGCAGTGCTTCTAGGC CGTGACACACCACAAGGGCTTAGG

*Nppb* AGGCGAGACAAGGGAGAACA GGAGATCCATGCCGCAGA

*Myh7* CGGACCTTGGAAGACCAGAT GACAGCTCCCCATTCTCTGT

*Lcn2* ATGTCACCTCCATCCTGGTCAG GCCACTTGCACATTGTAGCTCTG

*Adgre1* CGTGTTGTTGGTGGCACTGTGA CCACATCAGTGTTCCAGGAGAC

*Pecam1* CCAAAGCCAGTAGCATCATGGTC GGATGGTGAAGTTGGCTACAGG

*Acta2* CTCTCTTCCAGCCATCTTTCAT TATAGGTGGTTTCGTGGATGC

*18s* TTCTGGCCAACGGTCTAGACAAC CCAGTGGTCTTGGTGTGCTGA
